# Complete heart block following anaphylactic reaction to computed tomography contrast agent: A case report

**DOI:** 10.5339/qmj.2025.63

**Published:** 2025-06-18

**Authors:** Mustafa Mahmood Eid

**Affiliations:** 1 Emergency Department, Tawam Hospital, Al Ain, United Arab Emirates *Email: Dr.mustafa191982@gmail.com

**Keywords:** complete heart block, contrast dye, syncope, anaphylactic reaction, case report

## Abstract

**Background:** A complete heart block (CHB) entails the total loss of atrioventricular conduction and the failure to transmit any supraventricular impulses to the ventricles. To date, there have been no reports of CHB following the injection of intravenous contrast dye for computerized tomography.

**Case Presentation:** This case study details a patient who experienced a CHB and a syncopal episode after receiving intravenous contrast for a chest computed tomography (CT) scan. The patient was treated successfully for the anaphylactic reaction with steroids and intravenous fluid, and the heart rate improved with atropine and transcutaneous pacing.

**Discussion:** Contrast agents can affect cardiac conduction and endothelial integrity through their ionic strength, osmolality, and the release of histamine. These factors, combined with localized ischemia and adenosine release, may disrupt ion flow, potentially leading to transient or permanent atrioventricular block.

**Conclusion:** Anaphylactic reactions to CT contrast agents can lead to life-threatening cardiovascular complications, as exemplified in this case. Timely recognition and management of anaphylaxis, along with close cardiac monitoring, are crucial in such situations. Clinicians should be vigilant regarding the potential for severe cardiac manifestations in susceptible patients experiencing contrast-induced anaphylactic reactions, and they should take appropriate measures to ensure optimal care and recovery.

## INTRODUCTION

A complete heart block (CHB) results in the total absence of atrioventricular (AV) conduction, preventing supraventricular impulses from transmitting to the ventricles. Ventricular escape, or a junctional rhythm, maintains a perfusing heartbeat. Additionally, ventricular standstill may occur in some patients, potentially causing syncope (if self-terminating) or sudden cardiac death (if prolonged).^1^

When utilizing X-ray imaging techniques, radiocontrast media are prescription medications that enhance the visibility of internal organs and structures. They can lead to adverse effects ranging from itchiness to situations posing a life-threatening risk.^2^

The United Arab Emirates (UAE) has made substantial investments in healthcare and education infrastructure, including state-of-the-art facilities and medical equipment, in alignment with its Vision 2030 plan.^3^

The public healthcare system in the UAE is relatively robust, with health authorities consistently prioritizing improvements in the quality of care. In 2014, the UAE government set strategic objectives to establish a world-class healthcare system by 2021. These initiatives, endorsed by the UAE President and supported by the Vice President, were incorporated into the country’s national agenda. Following these announcements, both public and private healthcare organizations began adopting international accreditation standards, particularly those of the Joint Commission International (JCI). Thus, the UAE now leads globally in the number of JCI-accredited healthcare organizations.^4^

Al-Ain is a growing city with a population of approximately 767,000 people, according to the latest data, and features two major governmental hospitals: Al-Ain Hospital and Tawam Hospital. These two hospitals are well-equipped, offering a comprehensive range of specialties, advanced imaging facilities, and state-of-the-art operating rooms.^5^

This case involves a patient who developed a CHB after receiving intravenous contrast for a chest computerized tomography scan.

Written consent was obtained from the patient to publish this medical case, the accompanying images, the use of the data, and the data that appear in the article.

## CASE PRESENTATION

**Figure 1:**
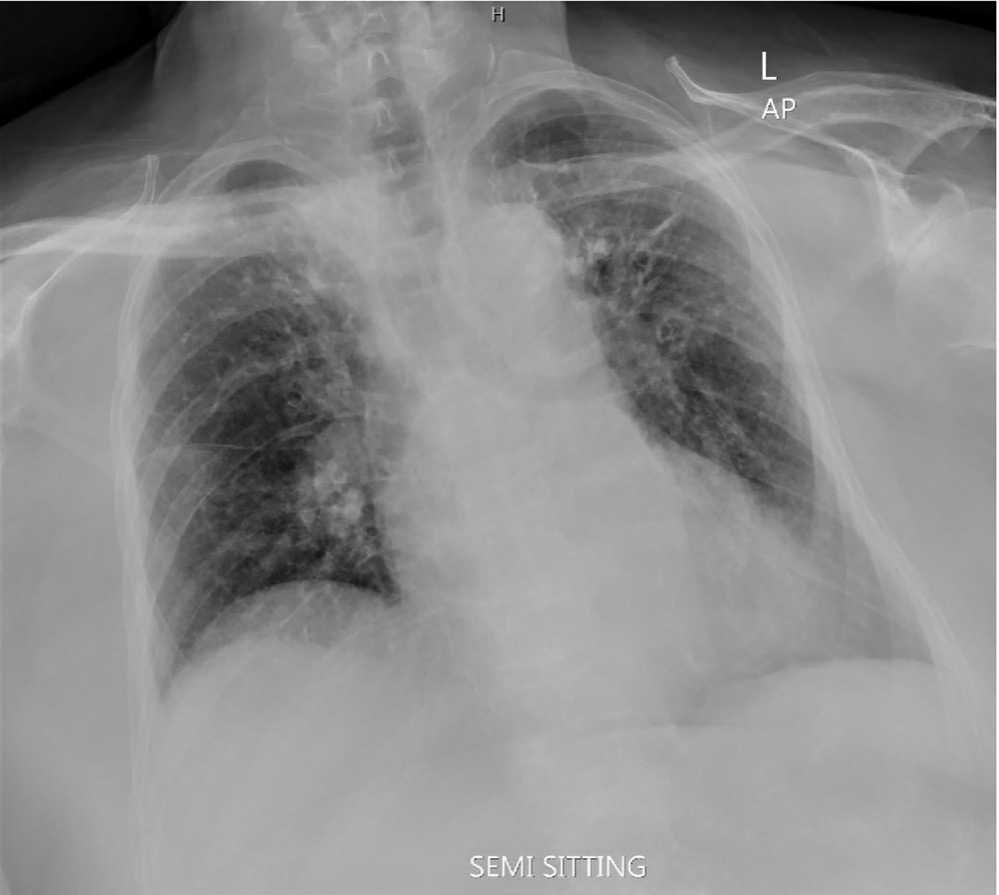
Chest X-ray showing bilateral accentuated bronchovascular markings with peribronchial mass and septal thickening (peribronchial cuffing). Additionally, there is a dilated, unfolded thoracic aorta with a prominent calcified aortic knuckle.

An 82-year-old female presented to the emergency department of a remote public hospital with a worsening productive cough over the past 3 days. The cough was accompanied by a change in sputum color from clear to yellow. She denied experiencing fever, chest pain, shortness of breath, vomiting, or diaphoresis. The patient has a known history of asthma, which coexists with chronic obstructive pulmonary disease. Moreover, she has diabetes, hypertension, and intermittent nocturnal obstructive sleep apnea. Her vital signs were within normal ranges. The chest examination revealed mild bilateral wheezes, and cardiac auscultation was unremarkable. Blood tests, including a complete blood count, renal function, and serum electrolytes, were all normal, except for an elevated C-reactive protein level of 55 mg/L (normal range: <9 mg/L). Her records revealed an electrocardiogram (ECG) showing features of left bundle branch block (LBBB), despite her denial of any prior cardiac events. A chest X-ray raised concerns about a possible lung mass (Figure 1).

As a result, a chest computerized tomography with contrast was arranged. The patient received a water-soluble, non-ionic iodine-containing contrast agent. The product’s name is Opitray 300 mg/mL. The patient received 80 mL of contrast at a rate of 2.5 mL/second, with a temperature of 37°C, through a peripheral venous line.

Unfortunately, after receiving the intravenous contrast, the patient immediately experienced a syncopal episode lasting approximately 10 seconds. She then regained consciousness, reporting that she was sweating and experiencing a sensation of burning all over.

**Figure 2:**
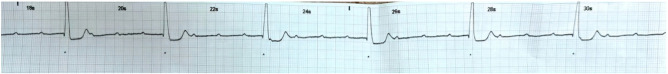
Patient’s ECG showing CHB with atrioventricular dissociation following the contrast die injection.

**Figure 3:**
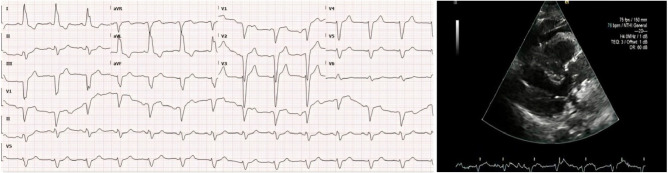
Patient’s ECG showing features of an LBBB in addition to a section of echocardiography following treatment.

The patient was promptly connected to a monitor, which revealed a heart rate of 25 beats per minute, a blood pressure of 60/30 mmHg, a bedside blood sugar level of 185 mg/dL, and an ECG displaying CHB (Figure 2). Consequently, the patient received a dose of atropine, methylprednisolone, and intravenous fluids within 5 minutes of the incident and was started on transcutaneous pacing within 15 minutes. Fortunately, the patient’s heart rate improved to 60 beats/minute (paced rhythm), and her blood pressure rose to 85/55 mmHg. She was subsequently transferred to a specialized hospital, where her condition further stabilized, with a blood pressure of 137/81 mmHg and a heart rate of 95 beats/minute. A follow-up ECG at the receiving hospital revealed a return to baseline, with the previously noted LBBB (Figure 3).

The patient was admitted for observation and further evaluation. The thyroid function test was reported within normal limits on the same day of admission, and an echocardiogram performed the day after revealed a normal-sized left ventricle with normal systolic function (ejection fraction of 60%). No intracardiac mass or shunt was detected, and there were no indications of pulmonary hypertension. There were no further episodes of heart block during her hospital stay. A respiratory panel tested positive for rhinovirus but negative for all other viruses, including influenza and SARS-CoV-2.

Consequently, the final diagnosis was CHB, likely caused by the contrast dye, in addition to viral bronchitis. The patient was discharged home after a 1-day admission, following an uneventful stay, for outpatient follow-up.

## DISCUSSION

A reaction to contrast dye may occur following diagnostic imaging tests such as MRI or computed tomography (CT) scans. This dye, typically iodine- or gadolinium-based, is administered intravenously to enhance the clarity of internal structures during imaging. Research indicates that serious allergic reactions to contrast dye occur in less than 1% of cases.^6^

Additionally, studies estimate the incidence of immediate reactions to nonionic contrast media ranges from 0.01% to 0.04% for severe reactions and up to 3% for mild reactions.^7^

Essentially, both Mobitz I and Mobitz II AV blocks can progress to CHB. In Mobitz I, CHB may result from the deteriorating condition of AV nodal cells, such as those affected by increased vagal tone during the acute phase of an inferior myocardial infarction. In Mobitz II, it may also be caused by the rapid onset of total conduction failure across the His-Purkinje circuit. This can be attributed to the progression of conduction system disease, resulting in genuine trifascicular block, or it can be secondary to septal infarction during an acute anterior myocardial infarction.^8^

Research has shown that earlier iterations of ionized contrast material directly block sinoatrial automaticity and AV conduction. This effect has been attributed to the ionic strength of the contrast material. Compounds with high osmolality are known to lengthen sinus cycles and extend AV nodal conduction.^9^

The exact causes of cardiac dysfunction associated with low-ionic-strength CT contrast agents are not fully understood and likely involve a combination of factors. When injected into the circulation, low-ionic contrast dye can lead to an increase in osmotic pressure, disrupting the regular flow of ions across the cell membranes of cardiac electrical cells. This disruption in ion flow can lead to changes in the heart’s electrical activity, including irregularities in the conduction of electrical impulses.^10^

Additionally, low-ionic contrast agents are known to cause less injury to endothelial cells compared to high-ionic contrast agents. This may limit the release of vasodilatory compounds and affect the heart’s natural ability to regulate blood flow. These factors can trigger a sequence of events that contribute to the development of CHB, resulting in a reduced oxygen supply to the heart muscle.^10^

The agent used in this case was a solution containing 636 mg of ioversol per mL, equivalent to 300 mg of iodine per mL. It had an osmolality of 645 mosmol/kg and a viscosity of 5.5 mPa·s at 37°C.

Furthermore, there is a hypothesis that the well-documented phenomenon of histamine release following the administration of radiocontrast material may be a contributing factor to AV block. Heller et al. observed a brief increase in histamine levels in the hearts of partially sensitized Guinea pigs after antigen challenge, leading to temporary AV block in some cases.^11^ This was later confirmed by Felix et al., who directly stimulated H1 histamine receptors in sensitized guinea pigs, resulting in various AV blocks.^12^

Another hypothesis suggests that the use of contrast media can lead to temporary ischemia due to blood depletion. In this scenario, a lack of oxygen exacerbates pre-existing vessel blockages, resulting in reduced overall blood flow and leading to abrupt ischemia. Local ischemia causes a reactive increase in endogenous adenosine, which may contribute to the development of transient third-degree AV block.^13^

To our knowledge, based on a narrative review, there are two reported case studies in the literature that describe heart block following contrast dye injection, unrelated to intracoronary injection. One describes a transient 2:1 AV block following intravenous contrast dye injection,^9^ and the other reports a CHB in a patient with a pre-existing Mobitz type 1 block who received intra-arterial contrast for femoral artery angioplasty.^13^ In our case, the patient was not taking any AV nodal blocking medications, had no previous history of cardiac issues despite her ECG showing an LBBB, and received intravenous contrast, which had not been previously reported.

## CONCLUSION

A contrast dye can rarely lead to a heart block, and most patients do not experience any adverse side effects. However, individuals with pre-existing cardiac conditions, patients with LBBB, especially elderly patients or those taking certain medications, may be more susceptible and may require increased monitoring before, during, and after imaging procedures involving contrast dye. Timely recognition and management of anaphylaxis, along with close cardiac monitoring, are crucial in such situations. Clinicians should be vigilant regarding the potential for severe cardiac manifestations in susceptible patients experiencing contrast-induced anaphylactic reactions, and they should take appropriate measures to ensure optimal care and recovery. Our patient was promptly treated with steroids, intravenous fluids, atropine, and transcutaneous pacing, leading to significant improvement and a positive response.

## Conflicts of interest

The authors have no conflicts of interest.

## Authors’ contribution

ME: conceptualization, methodology, software, validation, formal analysis, data curation, writing of original draft, writing of review and editing, visualization, supervision, and project administration.

## Ethical approval of studies and informed consent

This case complies with the guidelines for human studies and was conducted ethically in accordance with the Declaration of Helsinki. Ethical approval was not required according to hospital policies, as no experimental drugs or interventions were administered to humans or animals. Written patient consent was obtained from the patient to publish this medical case, the accompanying images, the use of the data, and the data that appear in the article.

## Acknowledgments

None.
